# Vitamin C May Increase the Recovery Rate of Outpatient Cases of SARS-CoV-2 Infection by 70%: Reanalysis of the COVID A to Z Randomized Clinical Trial

**DOI:** 10.3389/fimmu.2021.674681

**Published:** 2021-05-10

**Authors:** Harri Hemilä, Anitra Carr, Elizabeth Chalker

**Affiliations:** ^1^ Department of Public Health, University of Helsinki, Helsinki, Finland; ^2^ Department of Pathology and Biomedical Science, University of Otago, Christchurch, New Zealand; ^3^ School of Public Health, University of Sydney, Sydney, NSW, Australia

**Keywords:** ascorbic acid, COVID-19, common cold, quantile treatment effect, randomized controlled trials, SARS-CoV-2, statistics, treatment outcome

## Introduction

Interest in the potential effects of vitamin C for respiratory virus infections traces back to the 1940s (1–3). Numerous effects of vitamin C on the immune system have been demonstrated, which provide the rationale to investigate its effect on infections (4). The most comprehensive summary of the placebo-controlled trials on vitamin C and respiratory virus infections is the Cochrane review on vitamin C and the common cold (2).

The Cochrane review identified 31 comparisons that examined the effect of regular vitamin C supplementation of at least 0.2 g/day on 9745 episodes of respiratory virus infections occurring during the supplementation period. In adults the duration of colds was reduced by 7.7% (95% CI 3.7% to 11.8%, P = 0.00018) and in children by 14.2% (95% CI 7.3% to 21.1%, P = 0.000053) [2; Analysis 2.1]. These findings indicate that vitamin C has physiological effects, though the review did not demonstrate treatment effects on virus infections that had already started (2).

Based on two trials that compared two different vitamin C doses in separate trial arms (5, 6), a dose-response relationship for the effect of vitamin C was indicated, with a prediction that doses of 6-8 g/day may shorten viral upper respiratory infections by some 20% (2, 3, 7, 8).

Given the previous research on vitamin C, we read with great interest the report on the COVID A to Z Randomized Clinical Trial (COVID A to Z trial) which investigated the effects of 8 g/day vitamin C on the recovery from outpatient SARS-CoV-2 infection (9). However, we unfortunately found several methodological shortcomings in the trial. Here we describe our major concerns and statistical reanalysis of the findings. In addition to the usual care and the vitamin C arms, the trial included zinc, and zinc with vitamin C arms. These two arms are not considered in this commentary, however, major problems in the zinc intervention are described in the **Supplement**.

## Methodological Concerns With the COVID A to Z Trial

The COVID A to Z trial was “*stopped early for futility*” after recruitment of only 214 patients. The intention had been to recruit 520 patients (9). Stopping early seems unjustified.

The COVID A to Z trial authors described the rationalization for the sample size calculation as follows: “*We assumed that the usual care group would achieve a 50% reduction in symptom severity in a mean (SD) of 6 (3) days and that at least 1 of the other 3 study groups would achieve a 50% reduction in a mean (SD) of 5 (3) days*” (9). This indicates that the authors assumed a 1.0-day difference in time to a 50% reduction in symptom severity between the usual care arm and one of the intervention arms. This also indicates that a 1.0-day reduction was considered to be clinically important, otherwise sample size calculations would not have been based on this assumption.

The trial results were reported as follows: “*Patients who received usual care without supplementation achieved a 50% reduction in symptoms in a mean (SD) of 6.7 (4.4) days compared with a mean (SD) of 5.5 (3.7) days for patients receiving ascorbic acid, a mean (SD) of 5.9 (4.9) days for patients receiving zinc gluconate, and a mean (SD) of 5.5 (3.4) days for patients receiving both ascorbic acid and zinc gluconate supplementation*” (9).

Thus, time to a 50% reduction in symptom duration was 1.2 days shorter in the vitamin C arm than the usual care arm. Given that the observed vitamin C effect was 20% greater than the expected effect (1.2 vs. 1.0), it is illogical to have stopped the trial early because of “futility” (9). The authors do not explain this paradox: on the one hand, they considered that a 1.0-day reduction in symptom duration is a clinically important difference, yet on the other hand, the observed 1.2-day reduction was futile and justified early termination.

Another substantial concern with the COVID A to Z trial is that it was an “open label trial” (9) without a placebo administered to the control arm. Symptoms of respiratory virus infections are subjective and if there is no blinding there can be systematic bias in either direction between the treatment arms. If a patient believes that vitamin C is effective, he or she might under report symptoms. On the other hand, when patients are given an active intervention, such as vitamin C, they may observe and report symptoms at a lower threshold compared with the usual care arm. Given that the additional cost of a placebo in such a trial is marginal, it is surprising that none was used (9). Furthermore, it is not clear exactly what “open label trial” means and whether the patients were told what they were receiving and how that was communicated. The psychological effects can be quite different depending on how the intervention is described.

Our third concern is about the delay between the onset of symptoms and the initiation of vitamin C. For treating virus diseases, timing to initiation of treatment is important. For example, acyclovir and oseltamivir should be started as soon as possible after the first appearance of symptoms since a delay leads to less efficacy. Previously, long delays were proposed as a potential explanation for some therapeutic trials that did not find vitamin C effective (2, 7). In the Methods section, the COVID A to Z trial authors write: “*Patients were randomized … after a positive diagnosis*” (9). However, there is no description of the time between the onset of symptoms and the start of intervention. Given that the analysis was restricted to patients with confirmed COVID-19, it seems probable that the delay between the start of symptoms and the start of treatment was over 24 hours and may have been several days. It is unfortunate that this detail was not described (9).

Our fourth concern is about the distribution of vitamin C dosage: the trial report states “*8000 mg of ascorbic acid [was] (to be divided over 2-3 times per day with meals)*” (9). Intestinal absorption of vitamin C becomes saturated with high doses (10). Because of the decreased absorption, large single doses can cause stomach ailments. Hence, there is a big difference between administering 0.5 g/hour every hour and administering 4 grams twice a day. The total dose per day is the same, but the adverse effects may be much greater with the latter dosage. For example, in the large trial by Anderson et al. (5), the dosage was “*16 tablets (two every hour) on the first day of any illness*” (4). Thomas et al. provided no justification for their choice to administer vitamin C 2-3 times per day (9).

Our fifth concern is the lack of commentary around the baseline vitamin C status of the patients. There is great variation in dietary vitamin C intake and consequently in baseline plasma levels. In this respect vitamin C differs substantially from other drugs, for which the control group uniformly has baseline levels of zero. If baseline levels are high, then supplemental vitamin C is less likely to have an effect than when baseline levels are low (11).

## Reanalysis of the Reported Findings

The difference between the primary outcomes in the vitamin C and usual care patients was not analyzed appropriately for the COVID A to Z trial (9).

The report stated that the duration of symptoms, with the primary endpoint “*50% reduction in symptoms*”, was 6.7 days in the usual care arm and 5.5 days in the vitamin C arm (9). The 1.2-day difference was inappropriately ignored on the basis that the associated P-value was not less than 0.05 (12). The focus should be on the estimate and not on the P-value (13).

Moreover, given the great variation in the duration of untreated SARS-CoV-2 infection in the trial (from 2 days to over 3 weeks), the mean difference is a poor measure of the treatment effect. The 1.2-day reduction in symptom duration corresponds to an 18% reduction on the relative scale which is more informative (14). The 18% decrease is consistent with the predictions that 6-8 g/day of vitamin C will shorten viral respiratory infections by around 20% (2, 3, 7, 8).

Another approach to analyzing the effect of vitamin C on the recovery is to use survival analysis. This is not affected by the patients who do not recover during follow-up. The number of patients who recovered with the primary endpoint “*50% reduction in symptoms*” on a given day is published in Figure 3 of the report (9). We calculated the rate ratio of recovery between the vitamin C and usual care arms and found that vitamin C increased the rate of recovery by 70% (95% CI 6.8% to 170%, P = 0.025). This direct comparison of vitamin C and usual care arms was not published by Thomas et al. (9). Had the authors used appropriate statistical methods, they would have found a statistically significant difference. Our calculations and the redrawn survival curve are shown in the **Supplement**.

**Figure 1:**
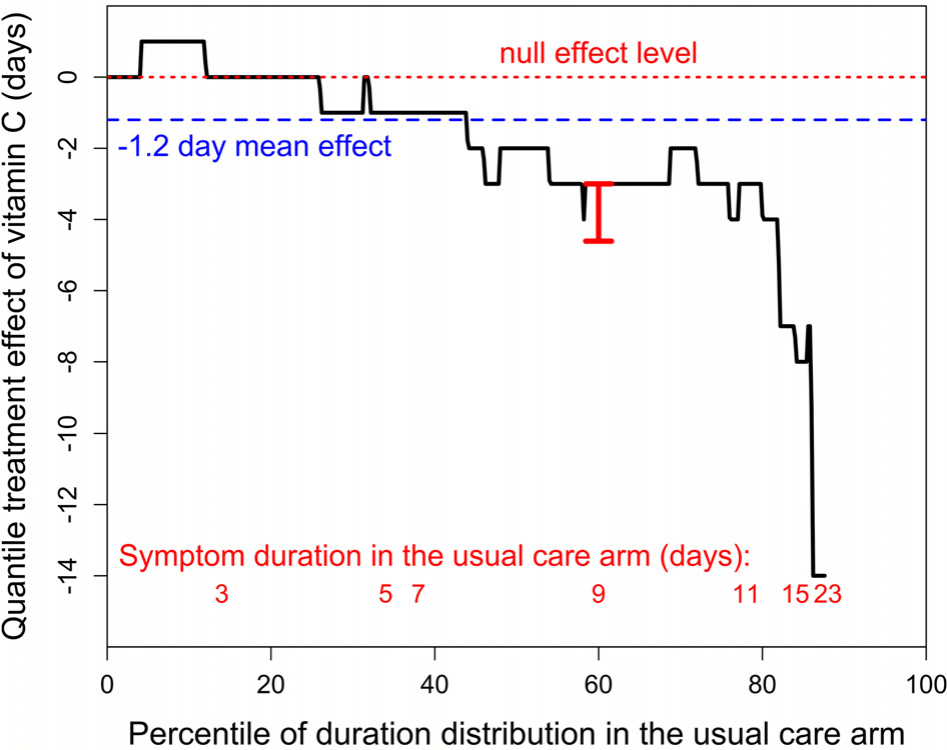
The quantile treatment effect (QTE) of 8 g/day of vitamin C on the duration of symptoms in outpatient cases of SARS-CoV-2 infection. The horizontal axis shows the distribution of the duration by percentiles. The black stepped line indicates the QTE of vitamin C. The horizontal red dotted line indicates the null effect. The blue dashed line shows the 1.2-day mean effect of vitamin C. The red vertical bar at the 60^th^ percentile indicates the 95% CI (-4.61 to -3 days; *t* = 7.3 P = 10^-10^), calculated with the *fit.crq* procedure of the R-statistical software (15–17). The red numbers at the bottom indicate the lowest percentile level for the odd number of days of duration of SARS-CoV-2 infection in the usual care arm. For example, 9-10 day infections cover the percentile range from 58^th^ to 76^th^, which corresponds to 9 patients, as the total number of usual care patients was 50. The QTE curve is stopped at the 88^th^ percentile since 6 patients in the usual care group were censored, i.e., did not recover by the end of the follow-up. See extracted data in **Table S1**, and our calculations and the redrawn recovery curve in the **Supplementary File**.

We also analyzed the effect of vitamin C by quantile regression as the quantile treatment effect (QTE) (15–17). In this approach, the distribution of symptom duration in the usual care arm is set on the horizontal axis as percentiles and the difference between the usual care and vitamin C arms is shown as the QTE on the vertical axis (**Figure 1**). The continuous black line indicates the QTE of vitamin C administration. For example, at the 60^th^ percentile level, duration was 9 days in the usual care arm, and 6 days in the vitamin C arm, which corresponds to a QTE of -3 days (95% CI -4.6 to -3 days; P < 0.001). The red dotted line indicates the null effect, and the blue dashed line indicates the −1.2 day day mean effect.

The QTE of vitamin C is heterogeneous over the distribution. In the 0 to 29^th^ percentile range, there is no indication of a difference between the arms with a mean duration of 2.9 days in the vitamin C arm and 2.7 days in the usual care arm. In the 30^th^ to 59^th^ percentile range, mean duration in the vitamin C arm was 1.6 days (26%) shorter (4.6 vs 6.2 days), and in the 60^th^ to 88^th^ percentile range, mean duration in the vitamin C arm was 4.0 days (34%) shorter (7.6 vs 11.6 days), see **Table S2** in the **Supplement**. Thus, the 1.2 day (18%) overall mean effect may be a substantial underestimate of the potential effect of vitamin C on longer cases of SARS-CoV-2 infection, which are of particular concern (18, 19). Vitamin C has also been shown to have a greater effect on more severely ill patients in some previous studies (20–23).

## Discussion

There is strong evidence that vitamin C can shorten the duration of respiratory virus infections (2, 3, 5–8). There are many different respiratory viruses, and their distribution varies over time and place. The respiratory viruses covered in many trials of vitamin C are non-specific and so it is unlikely that the benefits of vitamin C are constrained to a particular respiratory virus or virus group. Thus, it seems likely that vitamin C may also have an effect on the SARS-CoV-2 coronavirus (11, 24).

The COVID A to Z trial is important as it focused specifically on SARS-CoV-2 coronavirus patients and examined a high dose of vitamin C which was previously predicted to reduce the duration of respiratory virus infections by about 20% (2, 3, 7, 8). Unfortunately, there are several limitations in the trial methods and the trial was terminated early, even though the observed benefit from vitamin C was greater than assumed in the sample size calculations.

Regrettably, another recent trial on vitamin C published in JAMA also contained analysis errors (25), and another two recent reviews on vitamin C and respiratory infections were misleading (26, 27).

Our reanalysis of the COVID A to Z Trial results indicates that there is a statistically significant difference in the recovery rate between the vitamin C and usual care arms (P = 0.025), and the analysis of the quantile treatment effect indicates that there may be around 30% reduction in symptom duration in patients with the longest symptoms. These findings indicate a need for methodologically sound trials with larger numbers of patients to investigate the treatment effects of vitamin C against SARS-CoV-2.

## Author Contributions

HH originated the study, carried out the statistical analyses, and wrote the draft. AC and EC participated in the revision of the manuscript. All authors contributed to the article and approved the submitted version.

## Conflict of Interest

The authors declare that the research was conducted in the absence of any commercial or financial relationships that could be construed as a potential conflict of interest.
