# Supplementary material for: Vitamin C May Increase the Recovery Rate of Outpatient Cases of SARS-CoV-2 Infection by 70%: Reanalysis of the COVID A to Z Randomized Clinical Trial
Source: Front Immunol. 2021 May 10;12:674681. doi: 10.3389/fimmu.2021.674681 (PMC8141621; doi:10.3389/fimmu.2021.674681)
Supplement: Supplementary file 1 [file DataSheet_1.pdf]

Vitamin C may increase the recovery rate of outpatient cases of SARS-CoV-2 infection by 70%: reanalysis of the COVID A to Z Randomized Clinical Trial

Harri Hemilä 1), Anitra Carr 2), Elizabeth Chalker 3)
1. Department of Public Health, University of Helsinki, POB 41, Helsinki, Finland
2. Department of Pathology and Biomedical Science, University of Otago, Christchurch, New Zealand.
3. University of Sydney, Sydney, Australia

Harri Hemilä
http://orcid.org/0000-0002-4710-307X
https://www.mv.helsinki.fi/home/hemila/
harri.hemila@helsinki.fi

version 2021-2-28

Supplement to the manuscript published in Frontiers in Immunology
https://www.frontiersin.org/journals/immunology

This supplement describes the extraction of the rate of recovery of ambulatory patients with SARS-CoV-2 infection in the The COVID A to Z Randomized Clinical Trial [9], and the statistical reanalysis.

9. Thomas S, Patel D, Bittel B, Wolski K, Wang Q, Kumar A, Il'Giovine ZJ, Mehra R, McWilliams C, Nissen SE, Desai MY. Effect of High-Dose Zinc and Ascorbic Acid Supplementation vs Usual Care on Symptom Length and Reduction Among Ambulatory Patients With SARS-CoV-2 Infection: The COVID A to Z Randomized Clinical Trial. JAMA Netw Open. (2021) 4:e210369. https://doi.org/10.1001/jamanetworkopen.2021.0369

Contents **page**

Extraction of data on recovery from SARS-CoV-2 2
Table S1: Recovery from SARS-CoV-2 (from Figure 3 of [9]) 3
Recovery data in the R data set is consistent with the extracted spreadsheet data 4
Cox regression for vitamin C effect on recovery rate from SARS-CoV-2 5
Calculation of the 95% CI to the 60th percentage level 6
Figure S1: Redrawn survival curve for the effect of vitamin C on recovery from SARS-CoV-2 7
Table S2. Effect of vitamin C on symptom duration by percentile ranges 8
Problems in the zinc intervention in the COVID A to Z Randomized Clinical Trial 9

Extraction of data on recovery from SARS-CoV-2

The recovery data in the COVID A to Z Randomized Clinical Trial [9] was published as survival curve in Figure 3, which is copied below. When the number of patients is quite low, as in this case, it is possible to back-calculate from the survival curve the number of patients who recovered on each downward step. The size of the steps was measured from the digital figure as pixels and the scale of the figure as pixels was used to determine the number of recovered patients on each step. A spreadsheet was used to transform the pixel-values to the number of persons who recovered. Furthermore, the figure reported the number of patients still sick on each even day.

Figure 3 [9]

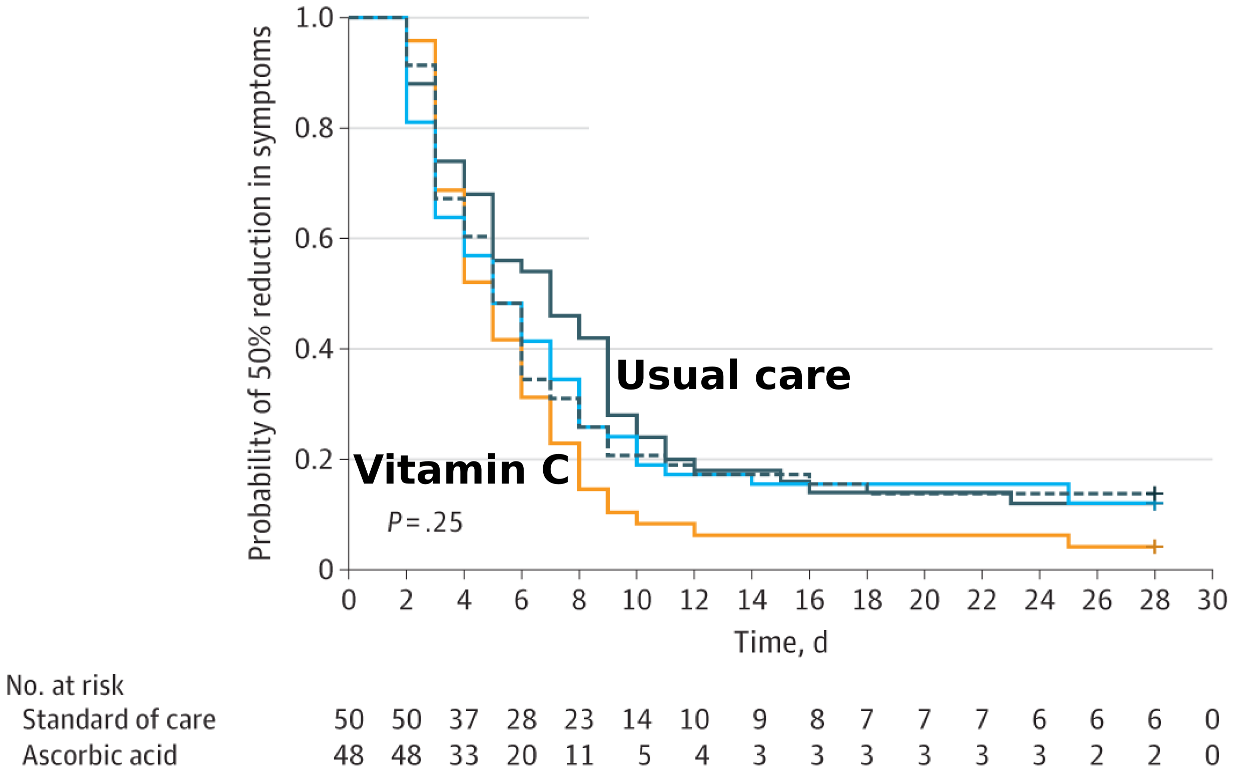

Table S1: Recovery from SARS-CoV-2 (from Figure 3 of [9])

The left-hand side of this table shows the measurement of the steps in Figure 3, seen previous page. The right-hand side shows the number of patients who recovered by the end of the given day. The number of patients on each step can be inferred with great accuracy. The number of patients reported on the even days in Figure 3 is consistent with our data extraction.

Extraction of recovery data from the COVID A to Z Randomized Clinical Trial report [9]

Still sick	Pixels
100%	228
0%	3812

The pixels are measured from the published Figure 3

Day	VitC N: 48 Pixels	Calculated	Published	VitC Cured	Day	Standard N: 50 Pixels	Calculated	Published	Standard Cured	Day
1	228	48,00	48		1	228	50,00	50		1
2	228	48,00	48	2	2	228	50,00	50	6	2
3	380	45,96		13	3	660	43,97		7	3
4	1348	33,00	33	8	4	1164	36,94	37	3	4
5	1944	25,02		5	5	1376	33,98		6	5
6	2324	19,93	20	5	6	1808	27,96	28	1	6
7	2696	14,95		4	7	1880	26,95		4	7
8	2992	10,98	11	4	8	2168	22,94	23	2	8
9	3292	6,96		2	9	2312	20,93		7	9
10	3440	4,98	5	1	10	2812	13,95	14	2	10
11	3516	3,96		0	11	2952	12,00		2	11
12	3516	3,96	4	1	12	3096	9,99	10	1	12
13	3592	2,95		0	13	3160	9,10		0	13
14	3592	2,95	3	0	14	3160	9,10	9	0	14
15	3592	2,95		0	15	3160	9,10		1	15
16	3592	2,95	3	0	16	3240	7,98	8	1	16
17	3592	2,95		0	17	3316	6,92		0	17
18	3592	2,95	3	0	18	3316	6,92	7	0	18
19	3592	2,95		0	19	3316	6,92		0	19
20	3592	2,95	3	0	20	3316	6,92	7	0	20
21	3592	2,95		0	21	3316	6,92		0	21
22	3592	2,95	3	0	22	3316	6,92	7	0	22
23	3592	2,95		0	23	3316	6,92		1	23
24	3592	2,95	3	0	24	3384	5,97	6	0	24
25	3592	2,95		1	25	3384	5,97		0	25
26	3668	1,93	2	0	26	3384	5,97	6	0	26
27	3668	1,93		0	27	3384	5,97		0	27
28	3668	1,93	2	2	28	3384	5,97	6	6	28
				28:Censored					28:Censored	

Recovery data in the R data set is consistent with the extracted spreadsheet data

```
> CrossTable(Thomas$Day, Thomas$vitC, prop.r ="F", prop.c ="F", prop.t ="F",
prop.chisq ="F")
```

```
Total Observations in Table:  98

             | Thomas$vitC
  Thomas$Day |         0 |         1 | Row Total |
-------------|-----------|-----------|-----------|
           2 |         6 |         2 |         8 |
-------------|-----------|-----------|-----------|
           3 |         7 |        13 |        20 |
-------------|-----------|-----------|-----------|
           4 |         3 |         8 |        11 |
-------------|-----------|-----------|-----------|
           5 |         6 |         5 |        11 |
-------------|-----------|-----------|-----------|
           6 |         1 |         5 |         6 |
-------------|-----------|-----------|-----------|
           7 |         4 |         4 |         8 |
-------------|-----------|-----------|-----------|
           8 |         2 |         4 |         6 |
-------------|-----------|-----------|-----------|
           9 |         7 |         2 |         9 |
-------------|-----------|-----------|-----------|
          10 |         2 |         1 |         3 |
-------------|-----------|-----------|-----------|
          11 |         2 |         0 |         2 |
-------------|-----------|-----------|-----------|
          12 |         1 |         1 |         2 |
-------------|-----------|-----------|-----------|
          15 |         1 |         0 |         1 |
-------------|-----------|-----------|-----------|
          16 |         1 |         0 |         1 |
-------------|-----------|-----------|-----------|
          23 |         1 |         0 |         1 |
-------------|-----------|-----------|-----------|
          25 |         0 |         1 |         1 |
-------------|-----------|-----------|-----------|
          28 |         6 |         2 |         8 |
-------------|-----------|-----------|-----------|
Column Total |        50 |        48 |        98 |
-------------|-----------|-----------|-----------|
```

```
> Thomas_S <- Surv(Thomas$Day, Thomas$Cured)
> Thomas_S
 [1]  2   2   3   3   3   3   3   3   3   3   3   3   3   3   3   4   4   4   4
4   4   4   4   5   5   5   5   5   6   6   6
[32]  6   6   7   7   7   7   8   8   8   8   9   9  10  12  25  28+ 28+  2   2
2   2   2   2   3   3   3   3   3   3   3   4
[63]  4   4   5   5   5   5   5   5   6   7   7   7   7   8   8   9   9   9   9
9   9   9  10  10  11  11  12  15  16  23  28+
[94] 28+ 28+ 28+ 28+ 28+
```

Cox regression for vitamin C effect on recovery rate from SARS-CoV-2

The survival analysis calculation was carried with the *coxph* program of the Survival package [S1] of the R statistical software [S2].

```
> Thomas_cox    <- coxph(Thomas_S ~ Thomas$vitC, method = "exact")
> summary(Thomas_cox)
Call:
coxph(formula = Thomas_S ~ Thomas$vitC, method = "exact")

  n= 98, number of events= 90

              coef exp(coef) se(coef)    z Pr(>|z|)
Thomas$vitC 0.5291    1.6974   0.2362 2.24   0.0251

            exp(coef) exp(-coef) lower .95 upper .95
Thomas$vitC     1.697     0.5891     1.068     2.697

Concordance= 0.559  (se = 0.032 )
Likelihood ratio test= 5.02  on 1 df,   p=0.03
Wald test            = 5.02  on 1 df,   p=0.03
Score (logrank) test = 5.09  on 1 df,   p=0.02

> lrtest(Thomas_cox)
Likelihood ratio test

Model 1: Thomas_S ~ Thomas$vitC
Model 2: Thomas_S ~ 1
  #Df  LogLik Df Chisq Pr(>Chisq)
1   1 -213.74
2   0 -216.25 -1 5.017     0.0251
```

References

S1. Therneau T (2020). A Package for Survival Analysis in R. R package version 3.2-3. https://CRAN.R-project.org/package=survival

S2. The R Project for Statistical Computing. https://www.r-project.org/

Calculation of the 95% CI to the 60th percentage level

The 95% CIs for the selected percentiles were calculated with the *crq* program of the quantreg package [16] of the R statistical software [S2].

```
> library(quantreg)
> fit.crq=crq(Thomas_S ~ Thomas$vitC, tau= 0.60, method="PengHuang")
> summary.crq(fit.crq, c(0.60,0.63,0.66,0.69), alpha = .05, R = 2000)

tau: [1] 0.6

Coefficients:
             Value  Lower Bd Upper Bd Std Error T Value Pr(>|t|)
(Intercept)  9.00  7.39      9.00      0.41      21.94    0.00
Thomas$vitC -3.00 -4.61     -3.00      0.41      -7.31    0.00

tau: [1] 0.63

Coefficients:
             Value     Lower Bd   Upper Bd   Std Error T Value   Pr(>|t|)
(Intercept)  9.00e+00  9.00e+00   9.00e+00   0.00e+00  1.96e+12  0.00e+00
Thomas$vitC -3.00e+00 -3.00e+00  -3.00e+00   0.00e+00 -8.02e+10  0.00e+00

tau: [1] 0.66

Coefficients:
             Value     Lower Bd   Upper Bd   Std Error T Value   Pr(>|t|)
(Intercept)  9.00e+00  9.00e+00   9.00e+00   0.00e+00  9.28e+11  0.00e+00
Thomas$vitC -3.00e+00 -3.00e+00   2.70e+00   1.45e+00 -2.06e+00  3.92e-02

tau: [1] 0.69

Coefficients:
             Value  Lower Bd Upper Bd Std Error T Value Pr(>|t|)
(Intercept)  9.000  9.000    13.094     1.044     8.618   0.000
Thomas$vitC -2.000 -6.094    -0.392     1.455    -1.375   0.169
```

References

16. Koenker R (2020). quantreg: Quantile Regression. R package version 5.67. https://CRAN.R-project.org/package=quantreg

S2. The R Project for Statistical Computing. https://www.r-project.org/

Figure S1: Redrawn survival curve for the effect of vitamin C on recovery from SARS-CoV-2

This survival curve is limited to the vitamin C and usual care arms shown in Figure 3 of the COVID A to Z Randomized Clinical Trial [9]. Compare with the modified Figure copied on page 2.
In the curves of the figure, the size of the steps downwards indicates the number of patients who recovered on the particular day. The red horizontal dotted lines indicate the 60th and 87th percentiles of the distribution of symptom duration, starting with the shortest colds from the top downwards. On the 60th percentile, the duration of symptoms was 9 days in the usual care group, and 6 days in the vitamin C group, corresponding to QTE of 3 days. On the 87th percentile, duration of symptoms was 15 days in the usual care group, and 8 days in the vitamin C group, corresponding to QTE of 7 days.

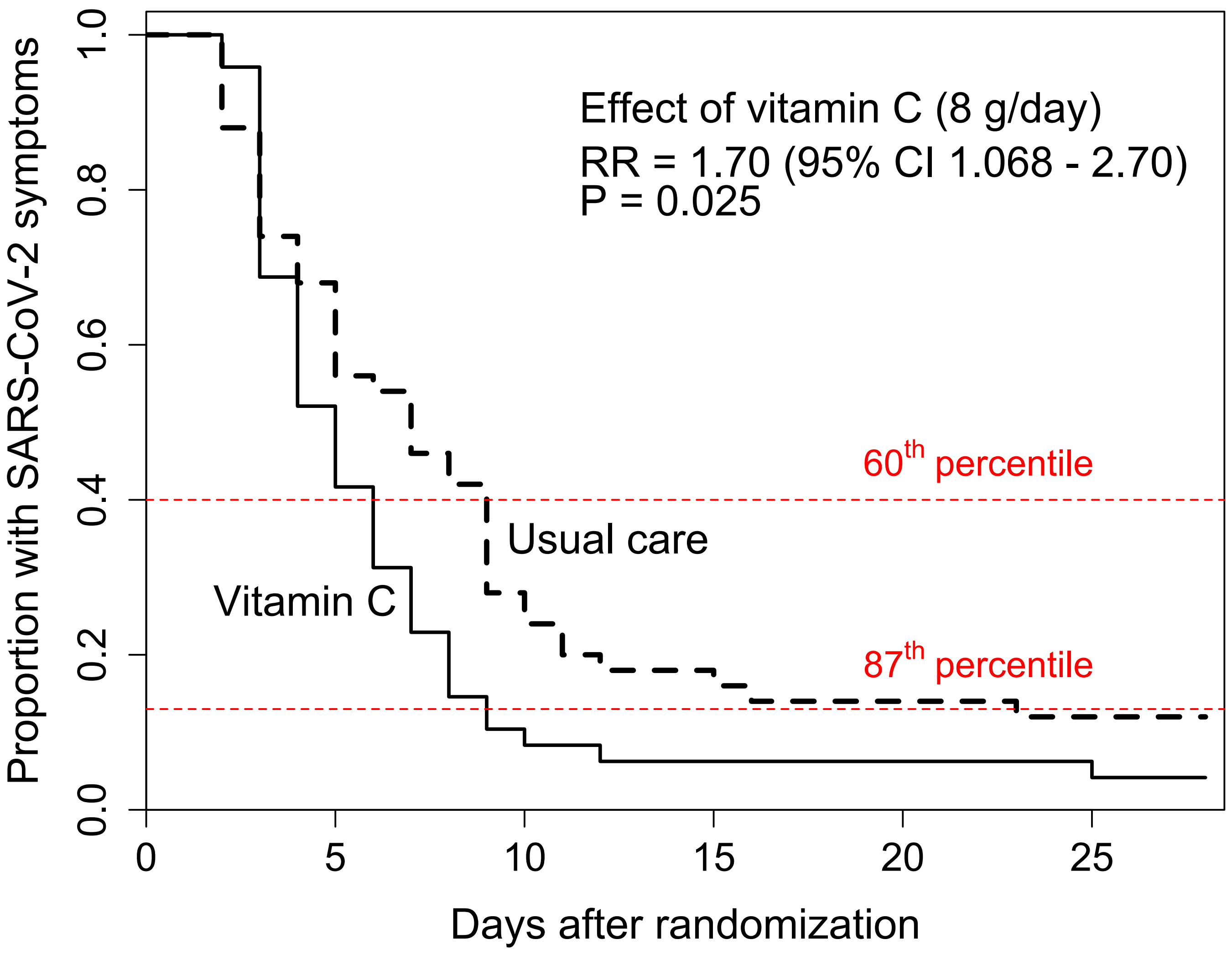

Table S2. Effect of vitamin C on symptom duration by percentile ranges

Percentile range	Mean duration of colds in the range		Effect of vitamin C	
	**Usual care**	**Vitamin C**	**Days difference**	**Percentage difference**
0 - 29	2.73	2.87	+0.13	+4.9%
30 - 59	6.20	4.60	-1.60	-25.8%
60 - 88	11.57	7.57	-4.00	-34.6%

There were 6 censored observations in the usual care group and therefore the analysis is cut at the 88th percentile level.

Problems in the zinc intervention in the COVID A to Z Randomized Clinical Trial

In addition to the usual care group and the vitamin C group, the COVID A to Z Randomized Clinical Trial included two further groups in their trial, a zinc gluconate group and a zinc gluconate together with vitamin C group [9].

Examination of zinc was motivated by three previous publications [S3-S5]. However, COVID A to Z report reference 6 (here reference S3) is the outdated version of the Cochrane review from 2011. The same Cochrane authors published a revised updated Cochrane review in 2013. However, the updated Cochrane review was retracted because of data plagiarism [S6,S7]. Therefore reference 6 [S3] should not be cited.

The two other papers were focused on zinc lozenges which are intended to be dissolved slowly in the mouth [S4,S5]. The COVID A to Z trial report does not clearly describe the type of tablet they used, but "*50 mg of zinc gluconate at bedtime*" [9] suggests that they administered a regular zinc tablet and not zinc lozenges. Furthermore, the RCTs that have found zinc lozenges to be beneficial, have administered the lozenges 6 times or more per day [S5]. A single dose at bedtime does not test the intervention that has been reported to be effective [S4,S5].

The first randomized trial on zinc lozenges used about 200 mg/day of elemental zinc [S4], but doses between 80 and 92 mg/day of elemental zinc were later found to be effective [S5]. The dosage published in the COVID A to Z trial of 50 mg/day of zinc gluconate [9] corresponds to just 7.2 mg/day of elemental zinc which is less than 1/10th of the dose of elemental zinc used in the lozenge trials which have reported benefit to common cold patients [S5]. A comment on the web page of the COVID A to Z trial claims that the trial used 50 mg/day elemental zinc and not the published 50 mg/day zinc gluconate [S8], so the reporting of the trial may be erroneous. Nevertheless, even if the dose was 50 mg/day elemental zinc, it is low compared with the trials in which zinc lozenges were shown to be effective [S4,S5,S9-S13]. The cited zinc lozenge references [S4,S5] do not provide any justification for choosing a single dose of 50 mg/day of zinc gluconate or elemental zinc at bedtime.

An individual patient data meta-analysis [S9] of three RCTs, in which 80–92 mg/day of elemental zinc was administered as zinc acetate lozenges to 199 common cold patients, calculated that zinc lozenges increased the rate of recovery by 215% (95% CI 110% to 370%, $P = 10^{-7}$). The most recent zinc acetate lozenge trial did not find benefit, which might be caused by low dose, rapidly dissolving lozenges, and short treatment [S14]. Nevertheless, for 2 days after the end of zinc/placebo use, the zinc participants recovered significantly slower compared with the placebo participants (p=0.003), which could be caused by the rebound effect so that the discontinuation of

the zinc lozenge treatment had a physiological effect in the harmful direction. Although that finding does not justify the usage of the particular lozenge, the finding is consistent with zinc lozenges having genuine effects on the common cold.

Further trials on zinc lozenges should test lozenges and protocols comparable with those used in the trials that reported benefit, and not ordinary zinc tablets administered once per day.

References to this section

S3. https://doi.org/10.1002/14651858.CD001364.pub3

S4. https://doi.org/10.1128/AAC.25.1.20
https://www.ncbi.nlm.nih.gov/pmc/articles/PMC185426

S5. https://doi.org/10.1177/2054270417694291
https://www.ncbi.nlm.nih.gov/pmc/articles/PMC5418896

S6. https://helda.helsinki.fi/handle/10138/153180

S7. https://doi.org/10.1002/14651858.CD001364.pub5

S8. Sickels M. 50mg elemental zinc was used [See Comment February 16, 2021 by M Sickels].
https://jamanetwork.com/journals/jamanetworkopen/fullarticle/2776305

S9. https://doi.org/10.1093/ofid/ofx059
https://www.ncbi.nlm.nih.gov/pmc/articles/PMC5410113

S10. https://doi.org/10.2174/1874306401105010051
https://www.ncbi.nlm.nih.gov/pmc/articles/PMC3136969

S11. https://doi.org/10.1186/s12875-015-0237-6
https://www.ncbi.nlm.nih.gov/pmc/articles/PMC4359576

S12. https://doi.org/10.1111/bcp.13057
https://www.ncbi.nlm.nih.gov/pmc/articles/PMC5061795

S13. https://doi.org/10.1016/j.mehy.2009.10.017
https://www.ncbi.nlm.nih.gov/pmc/articles/pmc7173295

S14. https://doi.org/10.1136/bmjopen-2019-031662
https://www.ncbi.nlm.nih.gov/pubmed/31980506
